# Modeling and Investigation of Deoxynivalenol Reduction in Wheat Flour After Cold Atmospheric Plasma Treatment Using Artificial Neural Networks

**DOI:** 10.3390/foods15030573

**Published:** 2026-02-05

**Authors:** Elizabet Janić Hajnal, Milan Vukić, Lato Pezo, Nenad Selaković, Nikola Škoro, Nevena Puač

**Affiliations:** 1 Institute of Food Technology, University of Novi Sad, 21000 Novi Sad, Serbia; 2 Faculty of Technology Zvornik, University of East Sarajevo, 75400 Zvornik, Bosnia and Herzegovina; milan.vukic@tfzv.ues.rs.ba; 3 Institute of General and Physical Chemistry, University of Belgrade, 11000 Belgrade, Serbia; latopezo@yahoo.co.uk; 4 Institute of Physics, University of Belgrade, 11080 Belgrade, Serbia; nele@ipb.ac.rs (N.S.); nskoro@ipb.ac.rs (N.Š.); nevena@ipb.ac.rs (N.P.)

**Keywords:** wheat flour, cold atmospheric plasma, deoxynivalenol reduction, HPLC-DAD, mathematical modelling

## Abstract

The aim of this study was to explore the effectiveness of cold atmospheric plasma (CAP) treatments for reducing the deoxynivalenol (DON) content in spiked white wheat flour samples containing 750 μg kg^−1^ DON. The flour samples were treated with plasma generated in air for durations of 30 s, 60 s, 90 s, 120 s, 150 s, and 180 s and at four distances from the cold plasma source: 6 mm, 21 mm, 36 mm, and 51 mm. An artificial neural network (ANN) model with three layers utilizing the Broyden–Fletcher–Goldfarb-Shanno (BFGS) iterative algorithm was developed to predict the reduction in deoxynivalenol (DON) content, moisture content, and temperature in wheat flour samples following cold atmospheric plasma (CAP) treatment. The model accounted for two key variables: the distance from the plasma source and the treatment duration. The ANN model exhibited excellent predictive performance, achieving coefficient of determination (*r*^2^) values of 0.999, 0.996, and 0.996 for DON reduction, moisture content, and temperature, respectively, during the training phase. The ANN model successfully identified the experimental optimal CAP conditions (51 mm distance and 150 s treatment), resulting in a 71% reduction in DON content. Multi-objective optimization (MOO) using the ANN further predicted the same level of reduction but at 168 s while maintaining acceptable moisture and temperature levels, representing the model-derived optimal treatment within the investigated design space. The study highlights the potential of ANNs to model complex relationships and optimize CAP treatment for efficient mycotoxin reduction in wheat flour.

## 1. Introduction

The most commonly found mycotoxin in wheat and wheat-based products worldwide is deoxynivalenol (DON), categorized as a type B trichothecene. DON, as a secondary metabolite mainly of *Fusarium culmorum* and *Fusarium graminearum*, represents a significant hazard to the food and feed processing chain, causing economic losses as well. Spoilage fungi in cereal crops can produce mycotoxins (secondary metabolites) under optimum conditions in the field or during storage, which can be toxic to humans and animals. Consumption of mycotoxin-contaminated food can cause hepatic, gastrointestinal, and carcinogenic diseases [1]. With over 140 identified fungal metabolites, *Fusarium* mycotoxins are the biggest class of mycotoxins. Numerous fungal species, primarily *Fusarium* (*F. graminearum* and *F. culmorum*), synthesize them [2]. Trichothecenes, especially DON, are prevalent in cereal crops and their concentrations very often exceed permissible levels [3]. DON contamination of wheat is widespread and frequently detected in the majority of samples tested globally [3]. Since wheat (*Triticum aestivum* L.) ranks as the second most produced grain crop worldwide, with about 800.79 million metric tons in 2024/25, its safety is of particular urgency [4].

DON is resistant to standard processes like milling, baking, and heating. Chemical reagents like ammonia, calcium hydroxide, chlorine, hydrochloric acid, ozone, sodium bisulfite, and sodium hydroxide can degrade DON, but none have been applied due to interference with standard grain processing or health hazards [2]. At the cellular level, DON has been found to have immunosuppressant or immune stimulatory effects depending on the dose and duration of exposure. DON is less toxic than other toxins, like T-2, but high doses can cause shock-like death [5,6,7]. Stimulatory effects are observed depending on the dose and duration of exposure. Symptoms include abdominal distress, increased salivation, malaise, diarrhea, emesis, and anorexia in sensitive species [6,8]. Chronic toxicity studies in experimental animals show decreased weight gain, anorexia, and altered nutritional efficiency. Animal species are sensitive to DON, with pigs being more sensitive than mice, poultry, and ruminants. In vivo, DON suppresses the immune response to pathogens and induces autoimmune-like effects similar to human immunoglobulin A (IgA) nephropathy [6,8].

In 1993, DON was placed in Group 3 (not classifiable as to its carcinogenicity to humans) of the International Agency for Research on Cancer [9]. Until July 2024, according to EU Commission Regulation No. 2023/915 [10], the maximum level for DON in cereals placed on the market for the final consumer, cereal flour, and semolina, bran, and germ as final products placed on the market for the final consumer was limited to 750 µg kg^−1^. The most recent Commission Regulation (EU) No. 2024/1022 [11] changed Regulation (EU) No. 2023/915 related to the maximum level of DON in certain foods. Now, in milling products of cereal, except for milling products of maize, its content is limited to 600 µg kg^−1^ [11]. Traditional technological processes for DON reduction may lead to food quality decline, but biodegradation and innovative processes and/or techniques offer mild conditions and high efficiency [12], which ensure the preservation of product quality with a significant reduction in DON content.

Among novel decontamination approaches, cold atmospheric plasma (CAP) has gained significant attention due to its ability to generate a broad range of reactive oxygen and nitrogen species (RONS) under mild, non-thermal conditions. CAP operates at atmospheric pressure, without any pumping facilities for pressure reduction, does not require chemical additives, and leaves no harmful residues, making it suitable for direct application to food products and simple integration into existing food processing systems. Unlike thermal, chemical, or UV-based treatments—which may compromise nutritional or sensory quality—CAP offers a residue-free and energy-efficient alternative that preserves the functional integrity of food matrices [13,14,15]. Furthermore, several recent studies have demonstrated its ability to effectively degrade DON and other mycotoxins in cereal-based systems through oxidation and structural disruption of toxic moieties [16,17].

Chemical processes induced by cold plasma have been studied extensively for many years in a wide range of applications, including recently established applications in plasma agriculture field and food technology [13,14,15,18]. Given these advantages, CAP has emerged as a promising tool for mycotoxin mitigation, particularly in food matrices where thermal or chemical treatments may be unsuitable. This is especially relevant for dry food powders like wheat flour, which presents a challenging matrix due to its heat-sensitive nutrients, the risk of denaturing functional gluten proteins, and the practical difficulty of treating a flowing, particulate material in vacuum. The underlying mechanism involves the interaction of plasma-generated reactive species—such as ozone (O_3_), nitric oxide (NO), and hydroxyl radicals (·OH)—with target contaminants, often resulting in molecular breakdown or functional group disruption. Due to their light mass, electrons in the gas are accelerated, gain high energies (1–10 eV), and are not in thermal equilibrium with other particles (ions, neutrals). The collisions of high-energy electrons with bulk gas molecules (N_2_, O_2_, and H_2_O) result in the formation of highly reactive short-lived (e.g., ·O, ·N, and ·OH) and long-lived species (O_3_, H_2_O_2_, NO, N_2_*, O_2_*, etc.) that can react with samples, performing processes of oxidation or reduction [19]. Considering the types of plasma sources, DBD sources are particularly convenient with respect to other atmospheric-pressure cold plasma sources as they can create large effective surfaces of active plasma, enabling treatment of larger areas. Electrode geometry in the case of surface DBD (SDBD) allows for the creation of a stable discharge close to the surface of the dielectric, which is not influenced much by the target type. Moreover, the source is modular, so many sources can be stacked together, increasing the effective surface for treatment.

The gas-phase plasma chemistry and reaction pathways that result in the formation of plasma-generated chemical species depend on various parameters, such as input power, gas mixture, sample distance, etc. The species created in CP operating at atmospheric pressure have very short mean free paths between collisions (order of μm); therefore, the plasma chemistry varies with the distance from the CP source. Determination of the precise plasma chemistry is a crucial input parameter for studying interaction processes and revealing the plasma mechanisms against particular pathogens. Mass spectrometry is a straightforward plasma diagnostic technique that can provide information on the created neutral and ionic species in plasma [20,21].

As many plasma decontamination studies have demonstrated that reactive oxygen and nitrogen species (RONS) generated by cold plasma sources play a crucial role in the interaction with pathogens [22], plasma diagnostics using mass spectrometry have focused on characterizing these species. As plasma technology begins to enter the food sector, this study presents findings on a promising new technique for mitigating mycotoxins. Although cold atmospheric plasma shows considerable potential as an innovative and effective approach for mycotoxin reduction, this technology is still under investigation and requires further validation and standardization before it can be considered ready for industrial application. To our knowledge, so far, there is no specific, universally established regulatory pathway for the use of plasma technology by food producers to reduce the mycotoxin content in food in most major jurisdictions, such as the United States (FDA), the European Union (EFSA), or other countries.

In years with severe DON contamination, conventional wheat processing steps may not be adequate to reduce toxin levels in white flour below the maximum allowed limit. Therefore, they may not ensure compliance under severe contamination. In a previous study, low-pressure RF helium and oxygen plasma for homologous wheat flour was studied, and the results indicated considerable reduction in DON content after 90 s and 150 s [23].

Artificial neural networks (ANNs) are increasingly used in food processing to model complex, nonlinear relationships between processing conditions and product responses [24,25]. For non-thermal technologies such as cold atmospheric plasma, where the treatment time and plasma–sample distance simultaneously affect reactive species generation, energy transfer, and mass and heat exchange, conventional modeling approaches are often inadequate [26]. ANN models provide an effective data-driven framework for capturing these interconnected effects and reliably predicting multiple responses, including contaminant degradation, moisture changes, and temperature variation [27,28,29].

In this study, we precisely determined the reactive species formed in plasma employing mass spectrometry and used ANN modeling for the first time to identify the CAP conditions that yielded the highest reduction in DON content. Therefore, the objective of this study was to investigate the possibility of predicting the reduction in DON content, moisture content, and temperature of wheat flour samples after cold atmospheric plasma treatment using a DBD source operating in air, based on the distance of the cold plasma source to the sample and process time. The study was performed using an artificial neural network (ANN) model with three layers and data obtained from mass spectrometry measurements. Various network topologies with 5 to 20 hidden neurons were evaluated over 100,000 training iterations, and the optimal configuration was selected by minimizing the validation error.

This study presents significant progress in mycotoxin mitigation for cereal processing, primarily through the first application of an artificial neural network (ANN) in modeling cold atmospheric plasma (CAP) treatment of deoxynivalenol (DON) in wheat flour. It addresses a significant gap in the literature by focusing directly on wheat flour, rather than on whole grains or liquid matrices. The research also includes a unique mechanistic analysis of reactive oxygen and nitrogen species using mass spectrometry, providing insights into the efficacy of plasma treatments. Furthermore, a genetic algorithm-based multi-objective optimization framework is utilized to enhance CAP treatment conditions, maximizing DON reduction while maintaining flour quality, thereby facilitating safer food processing practices.

To our knowledge, this is the first study to integrate predictive modeling, mechanistic diagnostics, and multi-objective optimization in the context of CAP-mediated mycotoxin (DON) reduction in wheat flour.

## 2. Materials and Methods

### 2.1. Material and Chemicals

For this study, white wheat flour was purchased at the market. The initial moisture content of white wheat flour was 14.1%. Before further procedures, a white wheat flour sample was analyzed to confirm that it was a blank sample without DON. To investigate the effect of atmospheric cold plasma on DON content, 10 g of white wheat flour was artificially contaminated, spiked, with DON standard solution (750 μg kg^−1^ in flour). This value was chosen to represent the maximum regulatory limit for DON in wheat flour as set by the European Union prior to July 2024 (Commission Regulation (EU) No. 2023/915) [10], and to reflect contamination levels commonly encountered in naturally contaminated samples. After spiking, the wheat flour sample was thoroughly mixed and then allowed to equilibrate at room temperature overnight to ensure homogenous distribution of DON within the matrix and to better mimic natural contamination scenarios. Subsequently, the spiked flour sample was stored at −20 °C until CAP treatment. The DON standard (10 mg) was purchased from Supelco^TM^ (Bellefonte, PA, USA). The stock solution was prepared by diluting the DON standard (100.1 µg mL^−1^) to a concentration of 10 µg mL^−1^ in a normal 10 mL vessel, from which further dilution series were made. Acetonitrile and methanol (all HPLC grade) were purchased from Merck (Darmstadt, Germany). Deionized water (Millipore, Bedford, MA, USA) was used for HPLC analysis.

### 2.2. Treating Spiked Samples with SDBD

#### 2.2.1. Plasma System for Treatment

**Figure 1:**
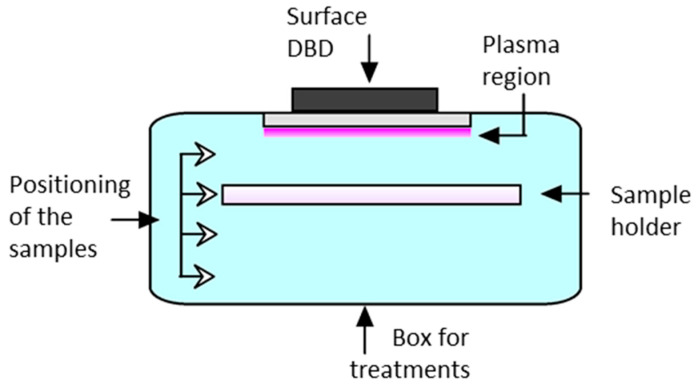
Schematic of the DBD plasma system for wheat flour treatment.

A schematic of the DBD plasma system for wheat flour treatment is shown in Figure 1. The DON-spiked wheat flour samples were kept in the sample holder during the treatments. The sample holder was positioned at four different distances from the plasma region.

The SDBD source was based on a 2 mm ceramic dielectric plate (length 70 mm × width 40 mm). The ceramic dielectric plate consisted of nine comb-like stripe electrodes (~1 mm width), with a 4 mm inter-stripe spacing on the bottom side and a conductive layer on the top side of the plate. Electrodes on both sides were connected to the HV transformer. We used a commercial HV transformer with a maximum output power of 85 W and floating signal output, so direct measurement of the voltage supplied to generate the plasma was not possible. A Variac regulator was used to control the input voltage to the transformer, and it was powered through the standard electrical grid at a frequency of 50 Hz. The output signal, supplied to the electrodes, was a high-frequency signal with several peaks 4–5 kV (peak-to-peak) modulated in continuous pulses at around 100 Hz. This enabled discharge ignition and stable operation. The detailed characteristics of the DBD system are described in more detail in ref. [30]. For the treatments, the SDBD source with an active cooler on the top side was mounted on the box lid. Spiked flour for treatment was weighed (10 g) in a Petri dish of 100 mm in diameter and placed in the box for treatment. The samples were spread in a thin layer (3 mm thickness) so that the screening effect and possible temperature gradients were minimized. For each plasma treatment, the distance between the flat dielectric surface of the SDBD emitter was precisely set to the required distance from the top surface of the flour layer inside the Petri dish. This was made possible by the compact dimensions of the plasma plate (70 mm × 40 mm), which fit within the Petri dish opening. Moreover, for further analysis, a complete amount of treated sample was homogenized and used to effectively average the effects of the treatment. The two-factor design assessed distance, which governed reactive species flux, and time, which determined the treatment dose, as key parameters for a scalable process. Specifically, 4 combinations of distances, (6 mm, 21 mm, 36 mm, and 51 mm) and 6 time points (30, 60, 90, 120, 150, and 180 s) resulted in 24 experimental conditions. Distances were selected to cover the operational range of our plasma source, from the point of near contact (6 mm) to a distance where significant plasma effluent dissipation was observed (~51 mm). The range of treatment times from 30 s to 180 s was chosen based on preliminary trials to identify the optimal treatment window. Shorter times (<30 s) showed negligible decontamination, while times exceeding 180 s risked inducing undesirable physical changes due to the observed temperature rise and moisture loss in the flour. Our preliminary trials also showed that 30 s intervals provided sufficient resolution to capture kinetic trends.

Plasma was formed on the lower side of the SDBD source in air, uniformly covering the surface of the SDBD source for an input voltage of 200 V, which was controlled by the Variac. The plasma thickness was around 1 mm, and it was checked visually that the operating regime was the same for all treatments. All plasma treatments were carried out under laboratory conditions, with a stable relative humidity of 45 ± 1%. Although the same humidity conditions were maintained for all experiments, minor fluctuations could not be entirely excluded. Since relative humidity may influence the plasma chemistry and the formation of reactive oxygen and nitrogen species, its potential impact represents a limitation of the present study and should be addressed in future investigations using controlled gas environments. However, as the humidity conditions were identical for all treatments, relative differences in the plasma chemistry and reactive species reaching the contaminated samples can be attributed primarily to the treatment distance and exposure time. After the treatments, the spiked samples were transferred from the box to sealed bags and stored at 4 °C until mycotoxin analysis.

#### 2.2.2. Mass Spectrometry

In this study, we employed mass spectrometry to investigate RONS formation in a cold atmospheric plasma system used for flour treatment. Specifically, the analysis of neutral species was performed using a commercial HIDEN Molecular Beam Mass Spectrometer (MBMS) HPR60 (Hiden Analytical Ltd., Warrington, UK). The unique design of this instrument enables direct sampling from atmospheric conditions, making it particularly suitable for the investigation of gas-phase plasma discharges and the identification of neutral species produced by DBD sources, as previously shown by Čech et al. [31]. To ensure accurate representation of the conditions used for plasma treatment process, the sampling orifice of the mass spectrometer was positioned at the same distance from the plasma source as the treated sample (6 mm, 21 mm, 36 mm, and 51 mm). The difference in the longest distance was because of experimental capabilities. Since we were not measuring absolute concentrations of the species but their time-resolved creation and development, this did not influence the results a great detail. Additionally, the discharge conditions during mass spectrometric measurements were maintained identical to those used during the plasma treatment of the sample—the input voltage on the voltage regulator was 200 V, while the total consumed power from the power grid was determined to be 76 W. RONS identification was carried out using the residual gas analyzer (RGA) mode of the MBMS in two different operational settings: (1) recording the integral spectrum of the plasma discharge to obtain a comprehensive overview of species present, and (2) the MID scan mode, which enabled real-time monitoring of radical evolution under both active and inactive discharge conditions. A shutter (swagelock-SL) in the inner side of the sampling orifice of the MBMS enabled closing the influx of particles from the surroundings and allowed us to measure the contribution of the signal from the species present only in the device itself. This distinguished the foreground and the background signals measured by the mass spectrometer. To ensure the reliability of the MS measurements, the instrument was tuned according to the manufacturer’s procedure prior to the beginning of the experimental series. Background spectra were recorded with the internal shutter closed and subtracted from all foreground signals to remove the signals of species from the unit itself. As the aim of the MS analysis was to follow the relative evolution of plasma-generated species rather than determine absolute concentrations, we did not perform absolute calibration. Measurements were repeated at least 3 times and they generated reproducible signals with sufficient signal-to-noise ratios. Signal intensities expressed in counts per second (c/s) correspond to the particular m/z value count rate registered by the detector.

In this study, mass spectrometry was employed as a diagnostic tool to qualitatively characterize plasma-generated reactive oxygen and nitrogen species and to monitor their relative evolution as a function of distance from the plasma source. The MS results were used to support the mechanistic interpretation of the observed DON reduction trends by identifying dominant long-lived reactive species and their relative changes under different treatment conditions. The MS analysis was not intended to provide absolute quantitative concentrations of species or to establish direct kinetic correlations with DON degradation.

### 2.3. Moisture Content

Moisture content in white wheat flour samples before and after the applied treatments was determined using an IM 9500 NIR instrument with the optional flour module (Perten Instruments, Hagersten, Sweden) and is expressed on a dry basis.

### 2.4. Quantification of DON Content Before and After Cold Atmospheric Plasma Treatment

#### 2.4.1. Sample Preparation

Sample preparation involved utilizing using MycoSep^®^225Trich SPE columns (Romer Lab, Inc., Union, MO, USA) for clean-up. Briefly, subsamples of spiked white wheat flour (5 g) underwent extraction with 20 mL of acetonitrile/deionized water (84:16, *v*/*v*) and were shaken for 30 min in a laboratory Griffin flask shaker (Griffin and George, Wembley, UK). The extracts were filtered through Whatman No. 4 filter paper (Whatman International Ltd., Maidstone, UK), and 5.0 mL of the filtrate was collected in the glass tube. The MycoSep clean-up column was employed to filter the extract upward, followed by transferring 2.0 mL of the upper layer into a glass cuvette for nitrogen evaporation (Reacti-Therm I#18821, Thermo Scientific, Bellefonte, PA, USA). The dry residue was dissolved in 0.40 mL of mobile phase and transferred to an HPLC vial through a regenerated cellulose (RC, 4 mm, 0.2 μm) premium syringe filter (Agilent Technologies, Strathaven, Lanarkshire, Scotland, UK).

#### 2.4.2. Instrumental Conditions and Method Performance

The DON content was determined using an Agilent 1260 Infinity HPLC system (Agilent Technologies Inc., Böblingen, Germany) consisting of a solvent degassing unit, a quaternary pump, an autosampler, a thermostated column, and a diode array detector (DAD). The DAD was set to 220 nm. Water/methanol/acetronitrile (90:5:5 *v*/*v*/*v*) at a flow rate of 0.60 mL min^–1^ under isocratic conditions were used as the mobile phase. The total run time was 25 min (15 min run, 10 min post-run). Separation was achieved using a Poroshell 120 EC-C18 column (4.6 × 100 mm, i.d. 2.57 µm) (Agilent Technologies Inc., Santa Clara, CA, USA) at 25 °C, and 15.0 µL standards and samples were injected into the duplicate. The retention time of DON was 3.80 min. The chromatograms were analyzed using Chemstation LC software ver. C.01.06 (Agilent Technologies Inc., Santa Clara, CA, USA). European Commission Regulation (EC) No. 401 [32] and Technical Report CEN/TR 16059 [33] from the European Committee for Standardization were utilized to establish and express validation parameters for the HPLC-DAD method. The method was validated concerning linearity, limit of quantification (LOQ), recovery, repeatability, and reproducibility with a standard curve obtained from duplicate injections at 5 concentrations ranging from 100 to 1000 ng mL^–1^ DON. The squared correlation coefficient (*r*^2^) was above 0.9990 for the calibration curve. For analytical methods to determine contaminants with prescribed maximum permitted concentrations, the LOQ should be well below the regulatory threshold. As per Technical Report CEN/TR16059 [33], the LOQ for deoxynivalenol (DON) must be 100 µg kg^−1^, which was confirmed by accuracy and repeatability tests on white wheat flour samples spiked to this level. The analytical method’s quality was evaluated using spiked white wheat flour samples, focusing on recovery, repeatability, and reproducibility. Recovery studies indicated high trueness, with values of 109.6%, 108.1%, and 107.8% for concentrations of 500, 750, and 1000 µg kg^–1^ DON, respectively. The relative standard deviation (%*RSDr*) was used to measure repeatability, and the results were 4.43%, 1.48%, and 1.36% at 500, 750, and 1000 µg kg^–1^ DON, respectively. The within-laboratory reproducibility (%*RSDR*) over three days yielded values of 8.38%, 7.69%, and 4.43%. The method met the criteria set by the European Official Decision procedure for confirmatory methods [32] and CEN/TR 16059 [33] from the European Committee for Standardization.

All flour samples were prepared and analyzed twice. The obtained results, i.e., concentration of DON before and after treatments, are expressed on a dry matter basis. The reduction in DON content was calculated as follows:

(1) $$Reduction\;of\;DON\;content\;\left(\%\right)=100-\left(C_{x}\cdot \frac{100}{C_{0}}\right)$$

where *C_x_* is the concentration of DON in the wheat flour sample after treatment, and *C*_0_ is the initial concentration of DON in the spiked wheat flour sample before treatment.

### 2.5. Mathematical Modelling

#### 2.5.1. Kinetics Modeling

To evaluate the deoxynivalenol reduction rate during cold atmospheric plasma treatment in wheat flour, kinetic modeling was performed. The temporal kinetics of the DON reduction rate, tested with different distances of the cold plasma source to the sample, were described using a four-parameter sigmoidal mathematical model (Equation (2)), which is highly suitable for biological systems. This model was used due to the complexity of the treated matrix (flour) and it was not related to plasma chemistry.

(2) $$y\left(t\right)=d_{1}+\frac{a_{1}-d_{1}}{1+{\left(\frac{t}{c_{1}}\right)}^{b_{1}}}$$

In Equation (1), the DON reduction rate (%) is represented as *y*(*t*), whereas the regression coefficients are denoted as follows: *a*_1_—minimum of the experimentally obtained values (at *t* = 0); *d*_1_—the maximally acquired value; *c*_1_—the inflection point (the point between *a*_1_ and *d*_1_); and *b*_1_—the Hill’s slope (the steepness of the inflection point *c*_1_).

#### 2.5.2. ANN Modeling

A three-layer multilayer perceptron (MLP) model—comprising input, hidden, and output layers—was used to develop an artificial neural network (ANN) for predicting the reduction in DON content, as well as the moisture content and temperature of wheat flour after treatment, based on the distance of the cold plasma source and process time. The ANN approach has been shown to effectively approximate nonlinear functions [34,35,36,37]. Prior to modeling, all input and output data were normalized to improve network performance. The input data were iteratively presented to the network [38,39], and the Broyden–Fletcher–Goldfarb–Shanno (BFGS) algorithm was applied to solve the unconstrained nonlinear optimization problem during ANN training.

The experimental database (72 data points from 24 plasma treatment conditions, each in duplicate) was randomly divided into training (60%), validation (20%), and testing (20%) sets. During model development, the number of hidden neurons (5–20) and the corresponding weight coefficients were optimized. To reduce dependence on random initialization, network training was repeated 100,000 times with different randomly assigned initial weights and biases, and the model with the lowest validation error was selected. The optimized ANN architecture consisted of a multi-layer perception with 8 input neurons, 11 hidden neurons, and 3 output neurons, resulting in 157 adjustable parameters (weights and biases). Although the dataset comprised 72 data points, overfitting was controlled through data normalization, early stopping, and independent testing. Model performance metrics and residual analysis confirmed adequate generalization within the studied experimental domain. Model robustness was assessed by 10-fold cross-validation and learning curves constructed from data subsets. Successful training was achieved when learning and validation errors converged toward zero, ensuring a conservative data-to-parameter ratio and minimizing overfitting.

Data acquisition for studying DON reduction in wheat flour after cold atmospheric plasma treatment was limited by the complexity, cost, and time required for each controlled experiment and chemical analysis. Despite the small dataset, the applied modeling strategy—with optimized network topology, extensive training, and cross-validation—ensured stable convergence and minimized overfitting.

Coefficients associated with the hidden layer (weights and biases) were grouped in matrices *W*_1_ and *B*_1_. Similarly, coefficients associated with the output layer were grouped in matrices *W*_2_ and *B*_2_. It was possible to represent the neural network by using matrix notation (*Y* is the matrix of the output variables, *f*_1_ and *f*_2_ are transfer functions in the hidden and output layers, respectively, and *X* is the matrix of input variables [40]:

(3) $$Y=f_{1}\left(W_{2}\cdot f_{2}\left(W_{1}\cdot X+B_{1}\right)+B_{2}\right)$$

Weight coefficients (elements of matrices *W*_1_ and *W*_2_) were determined during the ANN learning cycle, which updated them using optimization procedures to minimize the error between the network and experimental outputs [38,41,42], according to the sum of squares (SOS) and BFGS algorithm, and were used to speed up and stabilize convergence [43]. The coefficients of determination were used as parameters to check the performance of the obtained ANN model.

#### 2.5.3. Global Sensitivity Analysis

Yoon’s interpretation method was used to determine the relative influences of the reduction in DON content, moisture content of wheat flour samples after treatment, and temperature of wheat flour samples after treatment, based on the distance of the cold plasma source to the sample and process time [44]. This method was applied on the basis of the weight coefficients of the developed ANN:

(4) $$RI_{ij}\left(\%\right)=\frac{\sum_{k=0}^{n}\left(w_{ik}\cdot w_{kj}\right)}{\sum_{i=0}^{m}\left|\sum_{k=0}^{n}\left(w_{ik}\cdot w_{kj}\right)\right|}$$

Global sensitivity analysis was performed using Yoon’s interpretation method to assess the relative influence of the input factors, such as the distance of the plasma source (*d*) and treatment time (*t*), on the predicted outputs: DON reduction, moisture content (*MC*), and sample temperature (*T*). The method, based on ANN weight coefficients, provided a global measure of the contribution of each input parameter to the outputs, aiding in identifying the most influential variables for process optimization.

#### 2.5.4. Standard Score Calculation

Normal scores were calculated for each variable and were used for complex comparison of the observed samples, regarding the technological and chemical properties of the samples listed in Table 1. The ranking procedure between different samples was performed based on the ratio of raw data and extreme values for each applied assay [45], according to these equations:

(5) $$\bar{x_{i}}=1-\frac{\underset{i}{\max}\;x_{i}-x_{i}}{\underset{i}{\max}\;x_{i}-\underset{i}{\min}\;x_{i}},\;\forall i$$

in the case of “the higher, the better” criteria, or

(6) $$\bar{x_{i}}=\frac{\underset{i}{\max}\;x_{i}-x_{i}}{\underset{i}{\max}\;x_{i}-\underset{i}{\min}\;x_{i}},\;\forall i$$

in the case of “the lower, the better” criteria, where x_i_ represents the raw data.

#### 2.5.5. The Accuracy of the Model

The numerical verification of the developed model was tested using the coefficient of determination (*r*^2^), reduced chi-square (χ^2^), mean bias error (*MBE*), root mean square error (*RMSE*), and mean percentage error (*MPE*). These commonly used parameters can be calculated as follows [46]:

(7) $$\chi^{2}=\frac{\sum_{i=1}^{N}{\left(x_{\exp,i}-x_{pre,i}\right)}^{2}}{N-n},\;RMSE={\left[\frac{1}{N}\cdot \sum_{i=1}^{N}{\left(x_{pre,i}-x_{\exp,i}\right)}^{2}\right]}^{1/2},\;MBE=\frac{1}{N}\cdot \sum_{i=1}^{N}\left(x_{pre,i}-x_{\exp,i}\right),\;MPE=\frac{100}{N}\cdot \sum_{i=1}^{N}\left(\frac{\left|x_{pre,i}-x_{\exp,i}\right|}{x_{\exp,i}}\right)$$

where *x_exp,i_* denotes the experimental values; *x_pre,i_* indicates the predicted values calculated by the model; and *N* and *n* represent the number of observations and constants, respectively.

#### 2.5.6. Multi-Objective Optimization (MOO)

The developed ANN model was employed as a surrogate model in a multi-objective optimization (MOO) framework to determine the cold atmospheric plasma (CAP) operating conditions. The optimization problem was formulated to maximize the reduction in DON content while simultaneously minimizing moisture content and process temperature. Mathematically, the MOO problem can be expressed as follows:

- maximize *f*_1_(*x*) = DON reduction,
- minimize *f*_2_(*x*) = moisture,
- minimize *f*_3_(*x*) = temperature,

where *x* represents the vector of CAP process variables. The solution of the MOO problem is a Pareto front consisting of non-dominated solutions, where improvement in one objective cannot be achieved without deterioration in at least one other objective [47].

A genetic algorithm (GA) was applied to solve the MOO problem using a stochastic evolutionary approach based on selection, crossover, mutation, and inheritance operators [48]. The optimization was performed in MATLAB, ver. R2018b using the gamultiobj function. The initial population was randomly generated within the defined design space, and successive generations were obtained using non-dominated sorting and distance-based diversity preservation [47,49,50].

### 2.6. Statistical Analysis

The normality of the data distribution was evaluated using the Shapiro–Wilk test. Results showed that most variables did not significantly deviate from normality (*p* > 0.05). Data are expressed as mean values (*n* = 3). Differences between sample means were analyzed using Tukey’s HSD test. The statistical analysis was conducted using the STATISTICA V14.0.0.15 software package [51].

## 3. Results and Discussion

### 3.1. Mass Spectrometry of Plasma Source

**Figure 2:**
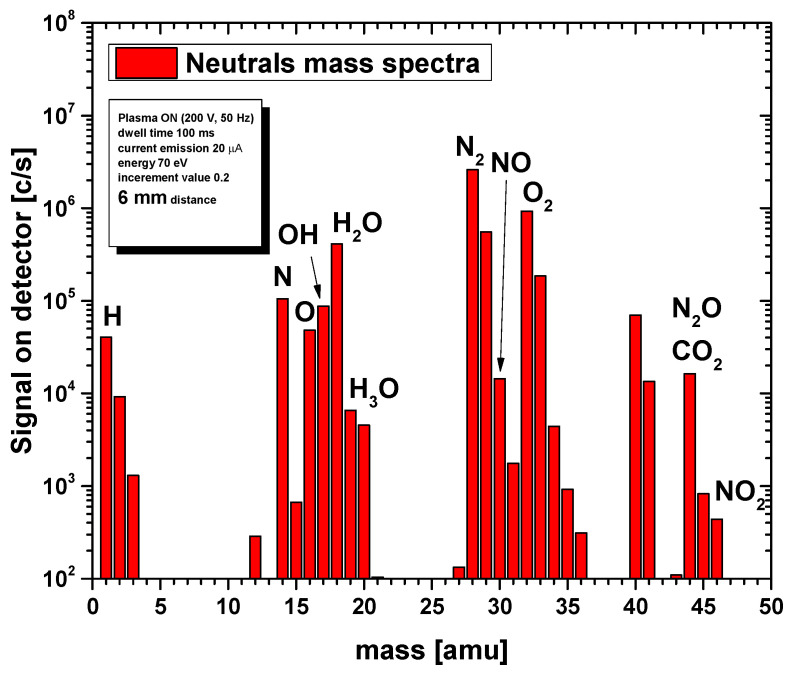
Complete neutral mass spectrum for 6 mm distance. Measurement was performed with the energy of electrons set to 70 eV in the ionization chamber. Data represent the averaged result of three independent measurements recorded under identical discharge conditions (*n* = 3).

In Figure 2, we present the integral mass spectrum recorded at a 6 mm distance from the plasma source. This spectrum, obtained using the RGA mode of the MBMS, provides an overview of all neutral species detected during operation of the plasma source in ambient air at 200 V input voltage controlled by the Variac. Apart from the species coming from the surrounding air, distinct alterations in RONS peaks were noticed in the rich spectrum of neutrals, e.g., NO (nitric oxide), NO_2_ (nitrogen dioxide), O_3_ (ozone), etc. When plasma is ignited, nitrous oxide is created in the plasma through the reaction $\cdot NO_{2}+\cdot N\to N_{2}O+O\cdot$ [52]. At *m*/*z* = 44, the detected signal corresponded to the combined contribution of N_2_O and CO_2_, as these species share the same molecular mass and cannot be distinguished by quadrupole mass spectrometry. Such overlap at *m*/*z* = 44 is a well-known limitation of residual gas and molecular beam mass spectrometry in atmospheric-pressure plasma diagnostics and has been reported in previous plasma chemistry studies [52,53,54]. Therefore, the recorded intensity at this mass-to-charge ratio represented the cumulative signal of both species rather than a single compound.

**Figure 3:**
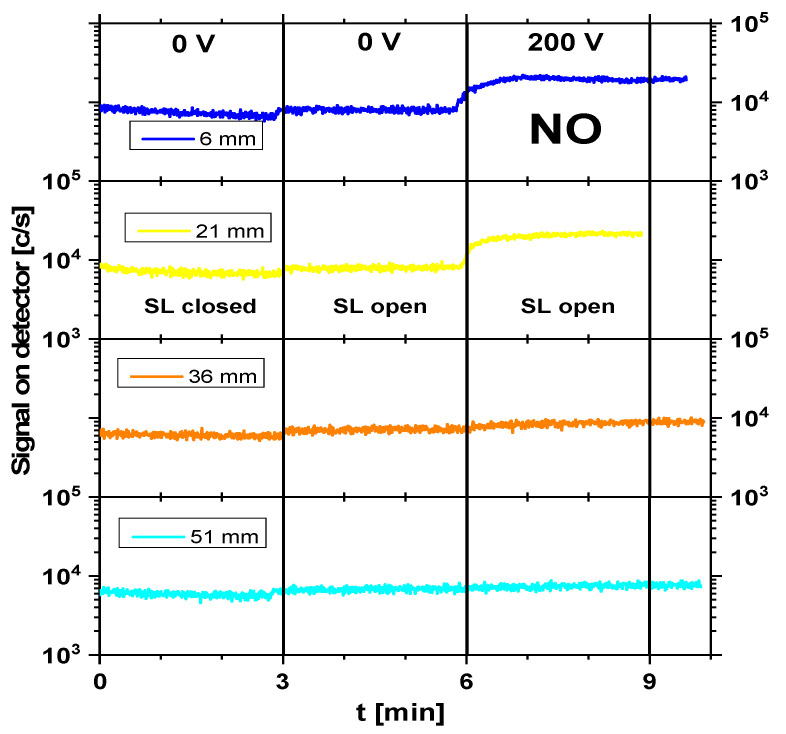
NO time evolution in cases of plasma-off (0 V) and plasma-on (200 V) conditions for different distances (6 mm, 21 mm, 36 mm, and 51 mm) from the plasma source to the mass spectrometer’s sampling orifice. SDBD was operating at 200 V input voltage. SL = internal shutter of the MBMS (open/closed).

**Figure 4:**
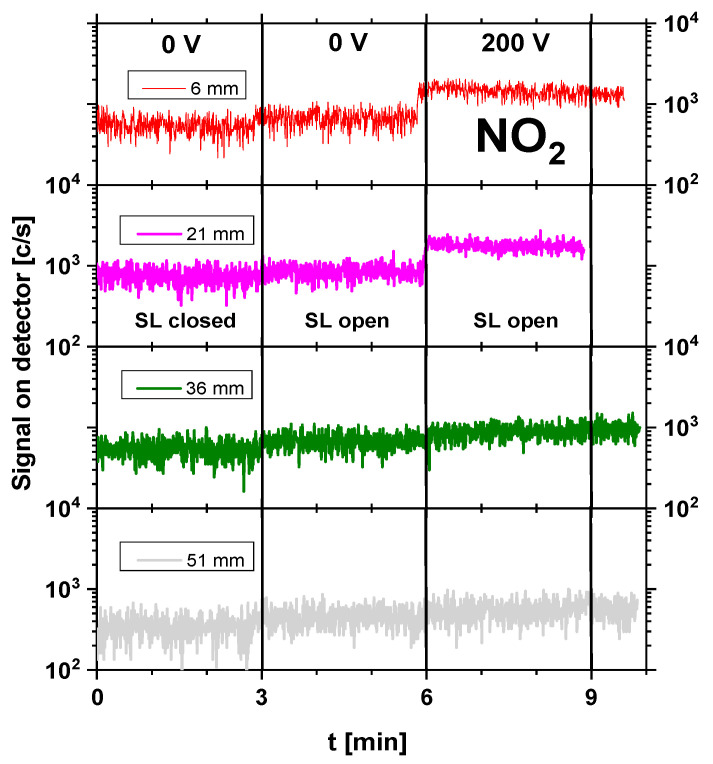
NO_2_ time evolution in cases of plasma-off (0 V) and plasma-on (200 V) conditions for different distances (6 mm, 21 mm, 36 mm, and 51 mm) from the plasma source to the mass spectrometer’s sampling orifice. SDBD was operating at 200 V input voltage. SL = internal shutter of the MBMS (open/closed).

**Figure 5:**
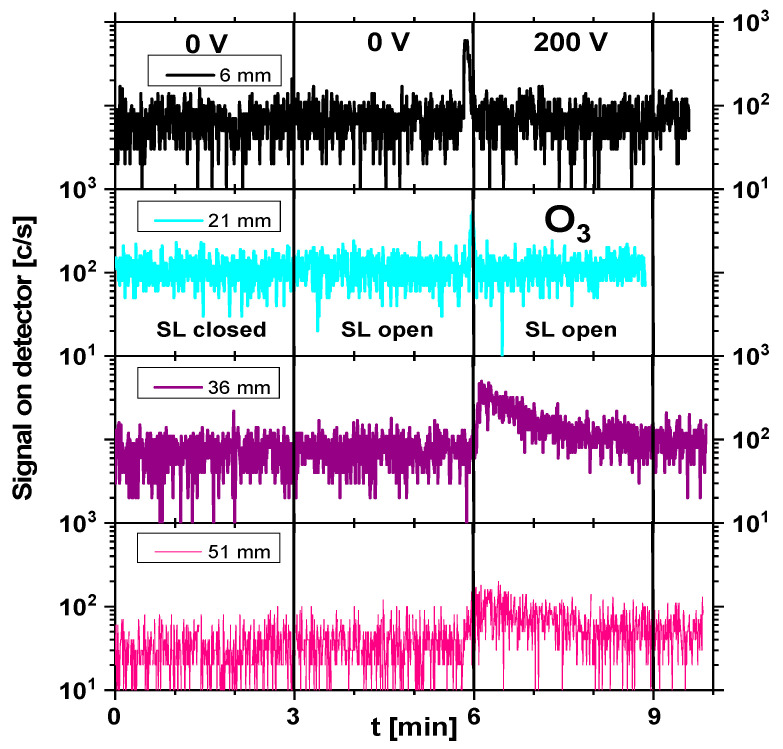
O_3_ time evolution in cases of plasma-off (0 V) and plasma-on (200 V) conditions for different distances (6 mm, 21 mm, 36 mm, and 51 mm) from the plasma source to the mass spectrometer’s sampling orifice. SDBD was operating at 200 V input voltage. SL = internal shutter of the MBMS (open/closed).

Although atomic species such as H, N, and O were detected in the mass spectrum, their presence was mainly attributed to fragmentation in the ionization chamber of the mass spectrometer. Due to their extremely short lifetimes and limited transport distances at atmospheric pressure, it was hypothesized that these short-lived species play a minor role in DON degradation under the applied treatment conditions. Instead, longer-lived reactive species were more likely to contribute to the observed effects. Therefore, further analysis focused on longer-lived, oxidizing species such as NO, NO_2_, and O_3_, which were more likely to contribute to chemical interactions with the wheat flour matrix. Figure 3, Figure 4 and Figure 5 illustrate the real-time evolution of NO, NO_2_, and O_3_ concentrations at different distances (6 mm, 21 mm, 36 mm, and 51 mm) between the plasma source and the mass spectrometer’s sampling orifice. The *y*-axis is given as counts per second (c/s), which is proportional to the concentration of particular species and enables relative comparison between concentrations. The black vertical lines on the graphs separate the different operating conditions under which the signals were recorded. In the graphs, one can distinguish between plasma-off (0 V) and plasma-on (200 V) conditions. Also, the mass spectrometer was operated in 2 regimes: SL open—when it recorded the sum of both foreground (outside the mass spectrometer) and background (inside the mass spectrometer) signals, and SL closed—when only the background signal was acquired. The duration of recording of the mass spectrometer for each operating condition was set to 3 min to allow enough time to stabilize after changing the conditions.

The NO signal (Figure 3) exhibited a strong dependence on the distance and plasma conditions. At 6 mm, the NO concentration was the highest, with an average value of around 19,000 c/s, indicating its primary formation near the plasma region. At 21 mm, the NO concentration reached its peak at around 20,100 c/s, suggesting a region of high NO stability before dilution began. As the distance increased, NO levels gradually decreased (8400 c/s at 36 mm and 7400 c/s at 51 mm), indicating dilution and oxidation into NO_2_. Under plasma-on conditions (200 V), the NO signal was significantly stronger compared to that under plasma-off conditions (0 V), confirming its production in the discharge.

The NO_2_ concentration followed an inverse trend to NO, increasing with distance (shown in Figure 4). At 6 mm, NO_2_ was present at 1500 c/s, while at 21 mm, its concentration increased to 1800 c/s, showing active oxidation of NO. At 36 mm, NO_2_ levels slightly decreased to 890 c/s, and at 51 mm, they further dropped to 560 c/s, likely due to secondary reactions reducing the NO_2_ concentration in the sampled region. The difference between SL open and SL closed signals confirmed that NO_2_ was actively formed in the plasma and not a background contaminant inside the spectrometer, particularly for the two shorter distances, which was apparent.

As shown in Figure 5, the O_3_ concentration in all cases exhibited a significant peak at the moment of plasma inception (point at 6 min after start of the recording). At that moment, there was a gradual increase in the voltage by using a potentiometer on the power supply, and within 10 s, plasma covered the whole surface of the SDBD source. This behavior suggested that ozone formation was initially enhanced at a lower plasma power, while increasing the voltage up to 200 V led to a stabilization effect. At larger distances (36 mm and 51 mm), the ozone levels were the highest, indicating that its formation was favored further from the plasma source, where three-body recombination reactions stabilized O_3_. Compared to Figure 3, which presents the NO signal, the measured intensities presented in Figure 4 and Figure 5 were of the same order or lower than the NO signal intensity. To be able to compare signals obtained for all three compounds, the settings of MS had to be the same. This led to a lower signal-to-noise ratio in the cases of NO_2_ and O_3_ molecules.

### 3.2. Reduction in DON Content by Atmospheric Cold Plasma Treatments

The results obtained through the analysis of DON content are presented in Table 1. Depending on the treatment applied, the reduction in DON content in wheat flour samples ranged from 33.1 ± 1.2% to 70 ± 4.4%. It can be observed that, by decreasing the distance of the plasma source from the flour sample and increasing the duration of treatment, the temperature increased while the moisture content in the flour decreased. The evaporated water present in the plasma-treated environment may theoretically participate in plasma-induced reactions, potentially leading to the formation of reactive species such as ·OH and H_2_O_2_, as suggested by established plasma chemistry mechanisms. In the present study, these species were not directly measured and are discussed here solely to support the theoretical interpretation of plasma–water interactions. Wheat kernels with optimal moisture content experienced more effective decreases in deoxynivalenol (DON) compared to drier kernels, demonstrating a factor that influences the degradation mechanism of DON [53].

Larger standard score (SS) values were assigned to more efficient DON reduction. SS analysis showed that the maximum reduction in DON content (71.0%) in wheat flour was obtained with the treatment performed at 51 mm for 150 s (Table 1). From the point of view of economic viability, the obtained optimal treatment that provided a satisfactory reduction rate (67.5%) of DON was achieved when wheat flour was treated for 60 s at a 36 mm distance from the plasma source.

The two main degradation pathways of mycotoxins induced by plasma are outlined in the literature. The double C=C bond undergoes addition reactions or it is reduced, and the lactone ring mainly undergoes ring opening and reduction of the carbonyl group. Additionally, reactions of oxidation, side chain shedding, and skeleton structure breaking are among the complex degradation mechanisms [54].

The specific formation pathways of DON degradation products and the mechanisms of each reaction site revealed that RONS mainly react with the C9=C10 double bond, the C12–C13 epoxy ring, and hydroxyl groups [55,56]. More specifically, O_3_ attacks the C9–C10 double bond and oxidizes the allylic carbon at C8 [57]. This is in agreement with the results of O_3_ concentrations in our study. At larger distances, ozone levels were the highest, and the treatment with the greatest DON reduction was at the distance of 51 mm. This effectively indicated that O_3_ exhibited a considerable effect on DON reduction, as the results of mass spectrometry of the plasma source indicated that its formation was favored further from the plasma source.

This study achieved a 71% reduction rate of DON after just 2.5 min at a distance of 51 mm from the cold plasma source. As previously noted, O_3_ levels were the highest at larger distances (36 mm and 51 mm), suggesting that its creation was more favorable farther away from the plasma source, where O_3_ was stabilized by three-body recombination processes. Based on the above, we assume that the degradation of DON at a 51 mm distance from the cold plasma source from the sample was favored by the oxidation process, since O_3_ is a strong oxidizing medium that can also cause the structure of DON to be destroyed [12]. Furthermore, Zhang et al. [54] identified ozone as a key oxidizing agent in their proposed degradation mechanism during DBD plasma treatment, confirming its role in disrupting the molecular structure of DON. These findings align with our results, where an increase in ozone levels corresponded to an increase in DON reduction.

The complex mixture of RONS generated by CAP, including hydroxyl radicals and ozone, leads to non-selective oxidation. Insights about the toxicity of the produced oxygenated compounds can be drawn from other advanced oxidation processes. For instance, a study on DON degradation by saturated aqueous ozone found that the resulting degradation products did not exhibit a significant change in overall toxicity compared to the parent DON molecule when assessed via in vitro cytotoxicity assays [58]. This suggests that the chemical structure of DON can be broken down by CAP, but also that the overall toxicological profile after CAP treatment may not be immediately exacerbated.

It is well-established that the reactive oxygen and nitrogen species (RONS) central to the degradation of deoxynivalenol (DON) can also initiate and propagate lipid oxidation in foods [59]. These effects of CAP also occur in wheat flour, leading to the oxidation of free fatty acids and phospholipids. However, it is noteworthy that at lower intensities of treatments, no significant lipid changes were detected [60]. CAP also can inactivate lipase and lipoxygenase in wheat flour, which can have beneficial effects [61]. All stated underscores the importance of process modulation to control changes in lipids in an intended direction.

Given that during this study neither the degradation products of DON nor the toxicity of the degradation products or changes in lipids were analyzed, future research should focus on these aspects.

### 3.3. Kinetics Model

**Figure 6:**
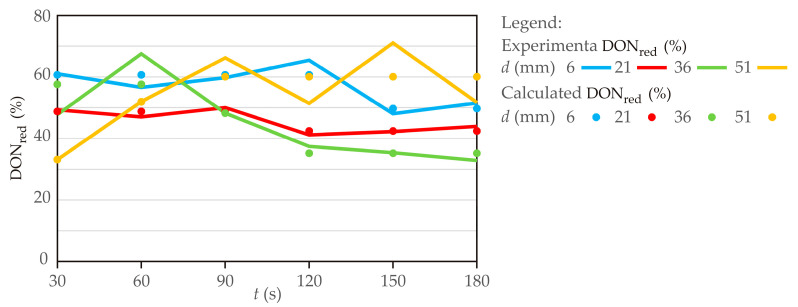
Kinetic modeling of deoxynivalenol reduction rate during cold atmospheric plasma treatment in wheat flour using different distances of the cold plasma source to the sample (points indicate calculated results, while lines represent experimentally obtained results).

The diagrams in Figure 6 depict the deoxynivalenol reduction rate during cold atmospheric plasma treatment in wheat flour using different distances of the cold plasma source to the sample, with data points representing sample treatment times from 30 to 180 s. The results demonstrate (Table 1, Figure 6) that both electrode distance (d) and treatment time (t) strongly influenced the efficiency of DON reduction in wheat flour subjected to cold atmospheric plasma treatment, with trends indicating a complex and nonlinear interaction between the two factors. At short distances (d = 6 mm), the plasma intensity was the highest and DON reduction generally improved with increasing treatment time, reaching a maximum at around 120 s (65.4%), after which efficiency declined, most likely due to the recombination of reactive species, energy saturation, or structural changes in the flour matrix that hindered further degradation.

At intermediate distances (21–36 mm), the reduction effect was noticeably lower (32–50%) and tended to decrease further with longer treatments, reflecting the weakening of plasma intensity as the distance increased. By contrast, at the largest electrode distance (d = 51 mm), the plasma effect became less predictable, with reductions ranging from only 33% at 30 s to the highest recorded value of 71% at 150 s, suggesting that extended exposure was necessary to compensate for reduced plasma density, although treatment efficiency became less stable. These findings emphasized that both underexposure (short time or large distance) and overexposure (excessive treatment times) can lead to suboptimal performance, and that an optimal treatment window exists. Overall, the data highlighted that effective DON degradation can be achieved through a balance of plasma intensity and duration, with the most favorable outcomes observed at moderate treatment times (90–150 s) combined with either short or long electrode distances, while intermediate distances appeared less effective. This behavior underscores the importance of carefully optimizing cold plasma parameters, as excessive treatment may compromise efficiency while unnecessarily increasing processing time and energy consumption.

Table 2 presents the kinetic parameters (*a*_1_, *b*_1_, *c*_1_, and *d*_1_) of a four-parameter sigmoidal model (Equation (2)) describing DON reduction as a function of the cold plasma source to the sample in millimeters. The upper asymptote *a*_1_, which denotes the highest predicted response level prior to the sigmoidal transition, decreased with increasing *d*_1_ parameter, indicating a reduction in DON content at greater distances, with the lowest value observed at *d*_1_ = 51 mm (33.1%). The inflection point *c*_1_, representing the midpoint of the transition, also declined (134.5 to 59.5), suggesting that the transition occurred earlier as the distance increased. Furthermore, the slope parameter *b*_1_ exhibited a decreasing trend (162.5 to 93.5), indicating a more gradual sigmoidal transition at greater distances. These findings suggested that DONred (%) was influenced by distance, potentially due to diffusion, degradation, or other spatial factors affecting its reduction.

The coefficient of determination (*r*^2^) was used to assess the goodness of fit of the four-parameter sigmoidal model in describing the temporal kinetics of DON reduction. At d = 21 mm, the highest *r*^2^ value (0.872) indicated the strongest agreement between the experimental data and the model predictions. By contrast, the lower *r*^2^ value observed at d = 51 mm (0.659) reflected a reduced ability of the model to capture the observed variability in the data. Intermediate *r*^2^ values at d = 6 mm (0.775) and d = 36 mm (0.749) indicated moderate model performance. The variation in *r*^2^ values across treatment distances suggested distance-dependent differences in model fit, without implying specific underlying mechanisms.

### 3.4. ANN Model Results

**Figure 7:**
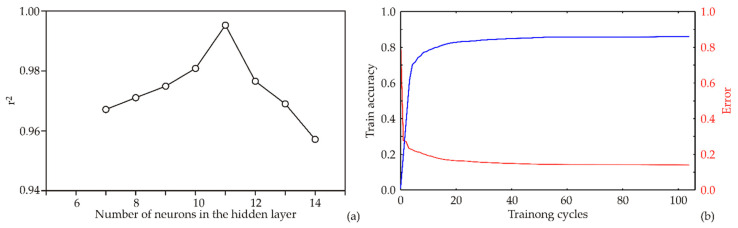
ANN calculation: (**a**) The dependence of the *r*^2^ value of the number of neurons in the hidden layer in the ANN model, (**b**) Training results per epoch.

The number of neurons in the hidden layer has a critical effect on model performance. To reduce the influence of random correlations caused by initial weight assumptions, each network topology was trained 100,000 times with different randomly initialized weights and biases, and the model with the lowest validation error was selected. This repetition was distinct from the number of training epochs in a single run. Among the tested configurations, the highest coefficient of determination (*r*^2^) during training was obtained with nine hidden neurons (Figure 7a). The model was trained for 100 epochs, and the corresponding performance metrics—training accuracy and error (loss)—are presented in Figure 7b. Accuracy consistently improved with increasing epochs and began to plateau between the 70th and 80th epoch, where the highest accuracy and lowest loss were recorded. After this point, only marginal gains in accuracy and minimal decreases in loss were observed, suggesting that extending training further would provide little benefit and could increase the risk of overfitting. Consequently, limiting training to approximately 70 epochs was considered optimal for achieving high accuracy while maintaining model generalizability (Figure 7b). After this point, only marginal gains in accuracy and further decreases in loss were observed, indicating the onset of overfitting. Consequently, limiting training to approximately 70 epochs was considered optimal for achieving high accuracy while minimizing the risk of overfitting (Figure 7b).

The acquired optimal neural network model showed a good generalization capability for the experimental data and could be used to accurately predict the reduction in DON content, moisture content of wheat flour samples after treatment, and temperature of wheat flour samples after treatment based on the distance of the cold plasma source to the sample and process time. Network MLP 8-11-3 obtained the highest values of *r*^2^ during the training cycle. The *r*^2^ values for the output variables were 0.999, 0.996, and 0.996, respectively (Table 3).

The obtained ANN model for the prediction of output variables was complex (157 weights–biases) because of the high nonlinearity of the observed system [62,63]. The slight inconsistency in *r*^2^ values among the predicted parameters likely reflected differences in the complexity of underlying relationships between input variables and each output. DON reduction, for instance, may be more directly influenced by distance and time than moisture content or temperature, which can be affected by additional uncontrolled factors (e.g., ambient humidity and sample variability). All *r*^2^ values exceeded 0.95, indicating excellent predictive performance of the ANN model across the evaluated datasets.

The goodness of fit between experimental measurements and model-calculated outputs, represented as ANN performance (sum of *r*^2^ between measured and calculated output variables), during the training, testing, and validation steps are shown in Table 4.

Artificial neural network (ANN) models demonstrate clear advantages over traditional kinetic models due to their ability to capture complex, nonlinear relationships between multiple input variables (treatment time and distance from plasma source) and output responses (DON reduction, moisture content, and temperature). Unlike kinetic models, which require predefined mathematical equations based on assumptions of reaction order or rate-limiting steps, ANN models learn directly from experimental data without assuming a specific functional form. The ANN model predicted the experimental variables reasonably well for a broad range of process variables. For the ANN model, the predicted values were very close to the measured values in most cases in terms of *r*^2^ values. The performance of the ANN model was evaluated using multiple goodness-of-fit metrics, including RMSE, MBE, MPE, SSE, AARD, and *r*^2^ (Table 4). These metrics quantify the deviation between predicted and experimental values and provide a statistically valid assessment of model accuracy and predictive performance. Comparisons to experimental error were removed, as SSE and other model-dependent metrics cannot be directly interpreted as experimental uncertainty. The ANN model had insignificant lack of fit tests, which meant that the model satisfactorily predicted output variables. A high *r*^2^ value was indicative that the variation was accounted for and that the data fit the proposed model satisfactorily [64,65].

The residuals from a fitted model were observed and the corresponding prediction of the response was computed using the ANN regression model. Residuals represent the differences between predicted and experimental values. Their random distribution around zero indicates that the unexplained variance behaves randomly and that no systematic patterns remain, confirming that the ANN model adequately captures the underlying relationship between the explanatory and response variables. The residuals appeared to behave randomly, which suggested that the model fit the data well (Table 5).

**Figure 8:**
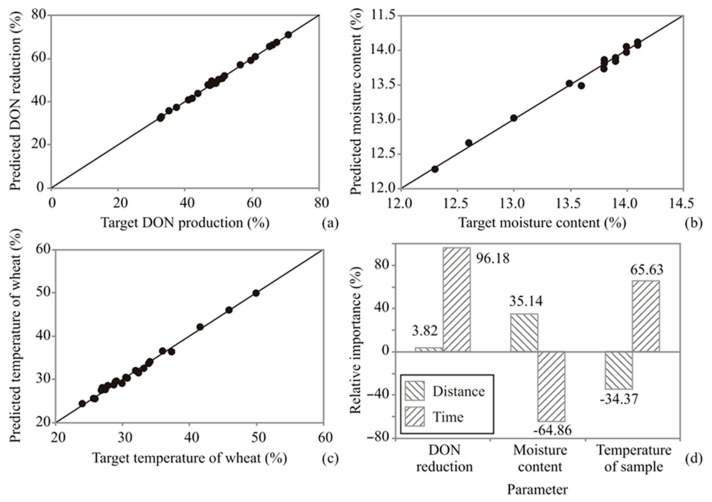
Experimentally measured and ANN model-predicted values of (**a**) reduction in DON content, (**b**) moisture of wheat flour samples after treatment, (**c**) temperature of wheat flour samples after treatment, and (**d**) the relative importance of input variables on outputs, determined using Yoon’s interpretation method.

Residual analysis of the developed model was also performed. Skewness measures the deviation of the distribution from normal symmetry. If the skewness is clearly different from zero, then the distribution is asymmetrical, while normal distributions are perfectly symmetrical. Kurtosis measures the “peakedness” of a distribution. If the Kurtosis is clearly different from zero, then the distribution is either flatter or more peaked than normal; the Kurtosis of a normal distribution is zero. A high *r*^2^ value indicates that a large proportion of the variance in the experimental data is explained by the model; however, satisfactory model performance also requires randomly distributed residuals, low RMSE, and unbiased MBE. Together, these metrics confirmed that the ANN model adequately captured the relationship between input and output variables [62,66]. The ANN model predicted experimental variables reasonably well for a broad range of process variables, as shown in Figure 8.

For the ANN model, the predicted values were very close to the measured values in most cases, in terms of *r*^2^ values. The SOS values obtained with the ANN model were of the same order of magnitude as experimental errors for output variables reported in the literature [38,42]. The ANN model had insignificant lack of fit tests, which meant that the model satisfactorily predicted output variables. A high *r*^2^ value was indicative that the variation was accounted for and that the data fit the proposed model satisfactorily [62,63].

In practical, real-world settings, however, several factors can affect the success the ANN prediction rate. While the ANN model provided accurate predictions under controlled experimental conditions, variations in the grain matrix (such as homogeneity of the sample, wheat variety, and initial DON levels), ambient environmental factors (relative humidity and temperature), and plasma treatment system stability (plasma intensity or uniformity) may lead to fluctuations in the effectiveness of CAP treatment. The ANN model can be expanded in future studies to predict reductions in the levels of various mycotoxins across different grains or food matrices, offering a generalized tool for optimizing plasma treatment. It could also be integrated into industrial-scale CAP treatments to ensure precise control over factors like plasma source distance and exposure time while maintaining effective mycotoxin degradation and product quality. Additionally, the model could be used in real-time monitoring systems for dynamic adjustments during production. Future research may incorporate variables like relative humidity or flour composition to improve the ANN model’s predictive accuracy and broaden its applicability. The ANN model could help identify optimal treatment conditions for different agricultural products, enhancing the versatility of CAP as a decontamination method.

#### 3.4.1. Global Sensitivity Analysis—Yoon’s Interpretation Method

In this section, the influence of input variables on the reduction in DON content, moisture content of wheat flour samples after treatment, and temperature of wheat flour samples after treatment, based on the distance of the cold plasma source to the sample and process time, was studied. The sensitivity analysis revealed that time was the predominant factor influencing all observed parameters, exerting a profound positive effect on DON reduction (96.18%) and sample temperature (65.63%), while showing a strong negative correlation with moisture content (−64.86%). Conversely, distance demonstrated a relatively minor influence, contributing most significantly to moisture content (35.14%) and temperature regulation (−34.37%) but remaining negligible (3.82%) in the context of DON degradation. These results indicated that while increasing processing duration is the most effective strategy for maximizing toxin reduction and thermal energy transfer, it simultaneously drives significant dehydration; meanwhile, adjusting the distance serves as a secondary control mechanism for modulating the sample’s physical state (moisture and heat) without substantially altering the efficacy of chemical reduction.

#### 3.4.2. Multi-Objective Optimization of the Outputs of the ANN

One of the main goals in this investigation was to maximize results, and these numerical tasks were solved using the MOO calculation in Matlab. The MOO procedure was defined by simultaneously maximizing reduction in DON content and minimizing moisture content and temperature in the ANN model.

The reduction in DON content is critical for improving flour safety, as high DON levels pose health risks. However, changes in moisture content and temperature during CAP treatment can influence flour quality parameters such as color and baking performance. Excessive drying or heating may negatively affect protein functionality or starch behavior. Thus, while maximizing DON reduction is desirable, it is essential to optimize CAP parameters to maintain desirable flour quality characteristics. Constraints used in the optimization procedure were applied within the experimental range of parameters. The number of generations reached 569 for the ANN model, while the size of the population was set to 200 for each input variable. The number of points on the Pareto front was 49 for the ANN model. The calculated maximum reduction in DON content during the plasma treatment was 71.0%, and the minimal moisture content and temperature were 12.5% and 30.7 °C, respectively. The distance was 51 mm, while the process time reached 168.4 s.

## 4. Conclusions

This study presents a novel application of CAP treatment of wheat flour, demonstrating significant DON reduction in the direct and static treatment of wheat flour as a powder matrix. The highest observed reduction in DON content (71%) occurred at a treatment distance of 51 mm from the plasma source, with a duration of 150 s. Under these conditions, the moisture content and the temperature of wheat flour samples after treatment were 13.9% and 30.8 °C, respectively. Mass spectrometry measurements of the plasma showed the presence of RONS in plasma. In particular, the creation and existence of O_3_, NO, and NO_2_ species at different distances between the active plasma zone (SDBD surface) and the flour sample were analyzed. As expected, increasing the distance between the plasma source and the sample reduced the concentration of RONS. However, the relative decrease was more pronounced for NO and NO_2_ species in comparison to O_3_. Additionally, O_3_ production rose during the transition period, when the input voltage is increased from zero to the operation voltage, thus resulting in higher overall production of ozone. The observed variability in reduction efficiency across different treatment times and distances suggests that matrix-related factors, such as moisture content and relative humidity, may also influence plasma efficacy. Additionally, since all samples in this study were spiked with a single DON concentration, further research is needed to determine whether the initial toxin level in naturally contaminated wheat flour affects reduction efficiency under plasma treatment. The artificial neural network model was shown to be adequate for the prediction of output variables (the *r*^2^ values during the training cycle for these variables were: 0.999, 0.996, and 0.996, respectively). By utilizing the ANN model, we were able to identify optimal conditions for maximizing DON reduction (71%) while maintaining acceptable flour quality. This highlights the advantages of ANNs in guiding experimental design and optimizing treatment parameters, which would be difficult to achieve using traditional experimental methods alone. While these findings are promising, the path to industrial application requires addressing clear limitations identified in this study. Future work must validate the treatment’s efficacy on naturally contaminated wheat flour, moving beyond the controlled model of artificially spiked samples used in this study. Although we monitored key RONS, a mechanistic validation of their role in the detoxification process and a comprehensive toxicological assessment of any resulting degradation products are needed to guarantee food safety. Finally, the influence of humidity, a factor not fully controlled in this study, must be taken into account and characterized, as it is an important factor affecting plasma chemistry. Therefore, for successful industrial-scale adoption of CAP technology, resolving these scientific questions is a prerequisite for tackling the broader regulatory and technical challenges in scaling CAP technology for the wheat processing industry.

## Figures and Tables

**Table 1:** The results of cold atmospheric plasma treatments on reduction in DON content in wheat flour.

	Inputs		Outputs			
Run	*d* (mm)	*t* (s)	DON_red_ (%) *	*MC* (%)	*T* (°C)	Standard Score
1	6	30	61.0 ± 1.6 ^ijk^	13.6 ± 0.2 ^ab^	37.4 ± 2.4 ^ij^	0.477
2	6	60	56.6 ± 2.3 ^hi^	14.1 ± 0.4 ^b^	29.0 ± 1.2 ^bcdef^	0.506
3	6	90	59.7 ± 3.1 ^ij^	13.8 ± 0.4 ^ab^	33.2 ± 1.0 ^fghi^	0.500
4	6	120	65.4 ± 1.1 ^jkl^	13.0 ± 0.5 ^ab^	41.6 ± 3.1 ^jk^	0.596
5	6	150	48.0 ± 1.6 ^efg^	12.6 ± 0.5 ^ab^	45.9 ± 0.6 ^kl^	0.463
6	6	180	51.5 ± 0.7 ^gh^	12.3 ± 0.2 ^a^	50.0 ± 3.0 ^l^	0.497
7	21	30	49.3 ± 3.5 ^fg^	14.1 ± 0.8 ^b^	27.0 ± 1.7 ^abc^	0.439
8	21	60	47.0 ± 0.7 ^defg^	14.0 ± 0.3 ^ab^	27.9 ± 1.2 ^abcde^	0.426
9	21	90	50.0 ± 2.1 ^fg^	14.0 ± 0.5 ^ab^	30.0 ± 1.2 ^bcdefg^	0.425
10	21	120	41.1 ± 1.6 ^bcd^	13.8 ± 0.4 ^ab^	32.0 ± 0.6 ^defgh^	0.359
11	21	150	42.2 ± 0.9 ^cde^	13.8 ± 0.5 ^ab^	34.1 ± 1.0 ^ghi^	0.342
12	21	180	43.9 ± 0.8 ^def^	13.5 ± 0.5 ^ab^	36.0 ± 0.8 ^hi^	0.388
13	36	30	47.7 ± 1.5 ^efg^	14.1 ± 0.7 ^b^	25.7 ± 0.8 ^ab^	0.441
14	36	60	67.5 ± 3.7 ^l^	14.1 ± 0.6 ^b^	26.9 ± 0.7 ^abc^	0.599
15	36	90	48.2 ± 1.1 ^efg^	14.1 ± 0.6 ^b^	28.7 ± 1.1 ^bcdef^	0.408
16	36	120	37.4 ± 0.5 ^abc^	14.1 ± 0.4 ^b^	30.5 ± 1.1 ^cdefg^	0.291
17	36	150	35.3 ± 1.1 ^ab^	13.9 ± 0.6 ^ab^	32.2 ± 1.9 ^efgh^	0.287
18	36	180	32.8 ± 0.6 ^a^	13.5 ± 0.9 ^ab^	34.0 ± 1.1 ^ghi^	0.316
19	51	30	33.1 ± 1.2 ^a^	14.1 ± 1.0 ^b^	24.0 ± 0.3 ^a^	0.336
20	51	60	51.9 ± 2.1 ^gh^	14.1 ± 0.6 ^b^	25.9 ± 0.6 ^ab^	0.476
21	51	90	66.2 ± 2.2 ^kl^	14.0 ± 0.4 ^ab^	27.5 ± 1.5 ^abcd^	0.598
22	51	120	51.4 ± 1.2 ^gh^	14.0 ± 0.1 ^ab^	29.1 ± 0.9 ^bcdef^	0.449
23	51	150	71.0 ± 4.4 ^l^	13.9 ± 1.1 ^ab^	30.8 ± 1.3 ^cdefg^	0.617
24	51	180	51.6 ± 1.7 ^gh^	13.9 ± 0.6 ^ab^	32.5 ± 1.2 ^fgh^	0.425
Polarity			+	-	-	

*d*—distance of the cold plasma source to the sample; *t*—treatment duration; *MC*—moisture content of wheat flour samples after treatment; *T*—temperature of wheat flour samples after treatment; DONred *—reduction in deoxynivalenol content; polarity—negative sign is associated with “the lower the better” criteria, while positive sign is associated with “the higher the better” criteria, as explained in Section 2.5.4. Values are calculated (see Section 2.5.4). Values designated by the same letter in a row are not significantly different (*p* > 0.05).

**Table 2:** Kinetics model parameters for a four-parameter sigmoidal mathematical model of deoxynivalenol reduction rate during cold atmospheric plasma treatment.

*d* (mm)	6	21	36	51
*d*_1_	49.7	42.4	35.2	60.0
*a*_1_	60.7	48.8	57.6	33.1
*c*_1_	134.5	103.9	90.3	59.5
*b*_1_	162.5	100.4	98.2	93.5
*r* ^2^	0.775	0.872	0.749	0.659

**Table 3:** Artificial neural network model summary (performance and errors), for training, testing, and validation cycles.

Network Name	Performance			Error			Training Algorithm	Error Function	Hidden Activation	Output Activation
	Train	Test	Valid	Train	Test	Valid				
MLP 8-11-3	0.997	0.998	0.999	0.275	0.189	0.203	BFGS 4179	SOS	Logistic	Identity

The performance term represents the coefficient of determination (*r*^2^), indicating the goodness of fit between experimental and predicted values. The error terms correspond to prediction error metrics (SOS), which quantify the deviation between model outputs and measured data and thus reflect the predictive accuracy of the ANN model.

**Table 4:** The “goodness of fit” tests for the developed ANN model.

Output Variable	χ^2^	RMSE	MBE	MPE	SSE	AARD	*r* ^2^
DON_red_	0.304	0.515	2.9 × 10^−5^	0.703	6.376	8.103	0.998
MC	0.002	0.040	2.9 × 10^−6^	0.231	0.039	1.660	0.993
T	0.324	0.533	−3.9 × 10^−5^	1.372	6.806	10.336	0.993

DON_red_—reduction of deoxynivalenol; MC—moisture content of wheat flour samples after treatment; T—temperature of wheat flour samples after treatment.

**Table 5:** The residual analysis tests for the developed ANN model.

Output Variable	Skew	Kurt	StDev	Var
DON_red_	−1.039	2.962	0.527	0.277
MC	0.665	1.069	0.041	0.002
T	0.237	−0.329	0.544	0.296

DONred—reduction in deoxynivalenol content; MC—moisture content of wheat flour samples after treatment; T—temperature of wheat flour samples after treatment.

## Data Availability

The original contributions presented in the study are included in the article; further inquiries can be directed to the corresponding author.

## Abbreviations

The following abbreviations are used in this manuscript:

DON	Deoxynivalenol
ANN	Artificial neural network
HPLC	High-performance liquid chromatography
DAD	Diode array detector
MOO	Multi-objective optimization
MLP	Multi-layer perceptron
